# 

**Published:** 1863-04

**Authors:** 


### CONTENTS FOR JANUARY.

Farmer Health—How best Secured; Eating—Rapidity, Frequency, Quantity; Catching Cold; Dress, Head-dress; Shoes, Corns; Housewifery; Potatoes; Baldness; Skating, etc.

### CONTENTS FOR FEBRUARY.

Premature Deaths; Success in Life; Responsibility of Writers; do. of Editors; Influence of Mothers; Controlling Temper; Our Daughters; Unskilled Labor; Farmers' Wives Overtaxed—in what Manner—the Remedy. Either Number sent post-paid for 15 cts.; both for 25 cts.

### CONTENTS FOR MARCH.

Dirt; Pulpit Power; Witnesses Three; Cherished Flower; Paine and Thorburn; Wire-workers; the Dying; Industrial Facts; Public Schools; the Irish; Cute Things; Ventilation; The One Spot.

### CONTENTS FOR APRIL.

Recreation; Interesting Facts; Reformers; John Randolph; Bible Confirmations; Paine and Lawrie Todd; Religious Newspapers; their Age; Sad Reflection; Henry Clay, etc., etc., etc.

